# How Brand Knowledge Affects Purchase Intentions in Fresh Food E-Commerce Platforms: The Serial Mediation Effect of Perceived Value and Brand Trust

**DOI:** 10.3390/bs13080672

**Published:** 2023-08-10

**Authors:** Shuai Ling, Can Zheng, Dongmin Cho

**Affiliations:** 1 Department of Design and Manufacturing Engineering, Jeonbuk National University, Jeonju 54896, Republic of Korea; shuailing@jbnu.ac.kr; 2 Department of Industrial Design, Jeonbuk National University, Jeonju 54896, Republic of Korea; mellgipson@jbnu.ac.kr

**Keywords:** fresh food, e-commerce platform, brand knowledge, perceived value, brand trust, purchase intention

## Abstract

The intense competition among fresh food e-commerce platforms in China has reduced the market share of the leading firms. This study aims to establish a model framework based on brand knowledge, perceived value, brand trust, and purchase intention to improve the market competitiveness of fresh food e-commerce platforms. Based on the analysis of 475 questionnaires using SmartPLS software, the results indicate that the established model framework provides an excellent explanation and forecasting (R^2^ = 45.5%) for consumers’ intentions to purchase fresh food. The path analysis results of this study show that there are significant positive effects among the model variables. Among antecedent variables, brand image has the greatest influence on perceived value, perceived value has the greatest influence on brand trust, and brand trust has the most significant impact on purchase intention. Furthermore, perceived value and brand trust have noteworthy mediating and serial mediating effects on brand knowledge and purchase intention. These findings have important implications for theoretical and managerial practices in the context of fresh food e-commerce platforms, providing insights on how to enhance customer purchase intentions.

## 1. Introduction

The gradual improvement of cold chain logistics supports the model of an enhanced quality of fresh food and reduced losses during transportation [1,2]. Therefore, the fresh food e-commerce platform (FFEP) is developing rapidly, and online purchases of fresh food have become the preferred choice for many people [3]. Recent research on the subject suggests that US consumers are progressively transitioning their fresh food purchasing habits towards e-commerce platforms [4]. According to the ‘Internet Economy 2020’ report, fresh food currently accounts for over 50% of total retail expenditure in Southeast Asia, with a projected increase to reach 40% of consumer spending on e-commerce platforms by 2030 [5]. According to recent data, the Chinese FFEP industry reached 311.74 billion yuan in 2021, representing a year-on-year (YoY) growth of 18.2% [2]. The worldwide outbreak of the global pandemic also resulted in an increase in online shopping for consumers in China [6]. By 2023, it is anticipated that the Chinese online fresh food market will have reached 419.83 billion yuan, underscoring the strong potential for development in the industry [2]. As a result, the online marketplace attracts many e-commerce brands, including Meituan Youxuan, Duo Duo Maicai, and Freshippo. This has brought over intense competition within the industry, as demonstrated by the 13.2% decrease in the revenue of the five largest online retailers of fresh food between 2018 and 2020 [7].

The development of China’s FFEP has followed a distinctive development model, largely driven by the influence of Internet giants such as Pinduoduo and Meituan. These companies have established sales networks centered around residential areas, implementing a community-based, localized, and personalized sales approach through direct engagement with community agents. This innovative operating model accelerated the development of FFEP, but it also caused serious problems. Certain FFEPs struggled to adapt to the rapid market development and, as a consequence of lacking competitive advantages and for various other factors, either had to shut down abruptly or were acquired by other companies. It is only a relatively small number of market-leading companies that managed to achieve profitability [8]. In addition to the rapid market development, there are two other reasons for this development: Fresh food is characterized by short timeliness and a high loss rate compared with traditional online sales products [9]. This not only increases the cost of sales for companies but also aggravates consumers’ concerns regarding the quality of the product. Additionally, the lack of positive word-of-mouth for domestic FFEPs has hindered the full realization of their brand effect. This not only affects the company’s reputation [10] but also influences consumers’ perception of fresh food, as they often rely on brands for quality and trust in their purchase decisions. As a result, this situation has hastened the closure of certain FFEPs [11,12]. In the sales process of fresh food, it is rare to identify products based on their brand, but FFEP provides an important bridge for consumers. Consequently, consumers no longer solely rely on traditional product brands when making purchasing decisions. Instead, they seek the brand effect of FFEP themselves. Therefore, understanding the impact of brands on consumers’ online fresh food purchases is crucial to enhance their willingness to buy. This holds significant importance for major fresh food e-commerce platforms to uphold their market competitiveness effectively.

Academics have conducted extensive research on the connection between FFEP and consumer purchase intentions. For instance, Lin et al. found that a platform’s quality of information, structure, and service can motivate consumers to make recurring transactions [13]. Wei et al. discovered that product information quality was not the main factor influencing customer buying decisions; the key factors emerged as price and quality [14]. Chen et al. identified that performance expectations and the social influence of e-commerce platforms could also prompt consumers to buy fresh food, with food safety awareness moderating its role [4]. Jin et al. indicated that diversity in the mix of certified and fresh food products increases consumer purchase intention [15]. Nonetheless, previous studies mainly focused on platform development and product attributes over the impact of the platform’s brand on consumer behavior, while brand knowledge should be equally valued as an essential factor in this regard [16,17]. Brand knowledge is the degree to which consumers are familiar with, recall, and identify a brand [18]. In this regard, perceived value and brand trust, two critical components that affect purchase intention, should also be taken into account [19,20]. Brand trust refers to how consumers perceive a brand as reliable [21]. The perceived value represents consumers’ comprehensive evaluation of gains and rewards in the consumption journey [22]. However, previous research has failed to consider the interaction and possible impact of brand knowledge with perceived value and brand trust in the context of fresh food online purchase intention. As e-commerce platforms continue to evolve, customers are increasingly exposed to a vast amount of information and navigate through complex psychological processes when making purchase decisions [23]. Therefore, this study poses the following research questions:

1. Can FFEP’s brand knowledge significantly influence consumers’ perceived value and brand trust?
2. To what extent do the consumers’ perceived value and brand trust impact consumers’ purchasing intention of buying fresh food in FFEP?
3. Do consumers’ perceived value and brand trust mediate the relationship between FFEP brand knowledge and fresh food purchase intention?

To address the limitations that previous studies on the subject had, this paper creates a research framework on how brand knowledge affects purchase intention. This study examines the relationships between the variables in the model and explores the potential serial mediation effects. This study addresses the research gap related to the FFEP brand effect on consumers’ purchase intentions for fresh food. It provides valuable practical insights for FFEPs aiming to enhance consumers’ purchase intentions and, in turn, strengthen their market competitiveness.

This paper is organized into the following sections. Section 1 focuses on the current status of FFEP and the main issues of the research. Section 2 focuses on this paper’s significant contributions and research hypotheses and establishes the research model through the hypotheses. Section 3 and Section 4 describe this paper’s research methodology and validation process. Section 5 and Section 6 discuss the research results and conclude. Section 7 addresses the limitations of this paper and outlines directions for improvement.

## 2. Research Hypotheses and Research Model

### 2.1. Research Hypotheses

#### 2.1.1. Brand Knowledge and Purchase Intention

Brand knowledge is the result of consumers’ processing of information regarding a particular brand [24,25]. It includes the various categories of brand-related information stored in a consumer’s recall and the network of associations that are formed based on this information [26]. Therefore, brand knowledge indicates the level of prominence a brand holds in consumers’ perceptions [27]. The foundation of brand equity is based on a company’s influence on consumers’ perceptions through marketing strategies to create brand knowledge [18]. Therefore, scholars consider brand knowledge a crucial component of brand equity and are offering it extensive evaluation [28,29,30].

According to Zenor and Hutchinson, brand knowledge includes brand familiarity and professional knowledge [31]. The brand knowledge summary model proposed by Keller describes the echelon relationship of brand knowledge and stipulates that brand knowledge includes two sub-components: brand awareness and brand image [29]. Brand awareness describes how deeply the brand is in consumers’ minds, while the brand image signifies consumers’ perception of the brand and is reflected in their association with the brand [32]. Keller’s study paved the way for future research in this field. In subsequent research, Keller refined the model and developed a comprehensive brand equity model that primarily relies on consumers’ knowledge structures [33]. Wang considers brand knowledge as the fundamental element of brand equity in terms of consumer perception. He divides the brand knowledge of Chinese consumers into four parts: quality, company reputation, image, and popularity [34]. In recent years, most researchers have initiated their studies based on Keller’s work, which categorizes brand knowledge into two dimensions: brand awareness and brand image [35,36].

In summary, this study suggests that consumers’ recognition and recall of the FFEP come from their perception of the brand awareness associated with the e-commerce platform. On the other hand, brand image perception reflects consumers’ evaluation of the recognizable aspects of the overall image, brand positioning, and market position of the platform in its external form. Therefore, the study examines both brand awareness and brand image as two crucial variables to investigate.

Brand knowledge is the measure of consumer recognition and familiarity with a brand [18]. Under suitable circumstances, a relevant recollection of brand information can result in positive attitudes, behaviors, and purchase decisions [37]. At the same time, consumers’ ability to recall and recognize brands on online sales platforms can reduce risk perceptions, which in turn increases the likelihood of purchase [38]. Brand awareness steers consumer perceptions and attitudes, serving as a potential driver for brand decisions [27,39]. In e-commerce, higher brand awareness increases the trustworthiness of information about a particular product on the brand’s platform, influencing purchase decisions [40]. This may be the case because a brand that lacks awareness is difficult to be considered as an option by consumers [41]. Brand image, seen as a determinant for purchase decisions in digital marketing [42], boosts brand loyalty and trustworthiness [43] and effectively minimizes the perceived risk associated with online purchases [44]. In online shopping for fresh food, brand image can have a potent halo effect, influencing consumers’ purchase intentions [45]. Consumer perception of the brand image creates a concrete and unique brand association that enhances consumers’ possibilities of the brand’s future adoption [46,47]. The following hypotheses are deduced from the analysis:

**Hypothesis** **1a** **(H1a).** *Brand awareness of the FFEP positively affects the purchase intention of fresh food.*

**Hypothesis** **1b** **(H1b).** *The brand image of the FFEP positively affects the purchase intention of fresh food.*

#### 2.1.2. Brand Knowledge and Perceived Value

Perceived value is an important link between consumers and businesses, especially throughout the various stages of the purchasing process [48,49]. If a product provides high levels of perceived value, it remarkably influences consumers’ willingness to purchase the product [50,51,52] and strongly affects their choices post-purchase [53]. Researchers have provided various explanations for this, including consumers’ assessments of the equilibrium between perceived product quality and price [54], evaluation of perceived quality [55], the comprehensive impression of the firm’s product information, service, and experience [56], the net benefits of gaining product value and incurring costs upon consumption [57], and trade-offs that are relatable to competitive products [58]. By analyzing and summarizing the different definitions of perceived value, researchers have also identified the following characteristics of perceived value; namely, subjectivity [59], hierarchical [60], and dynamicity [61]. In the dimensional division of perceived value, scholars in recent years have mainly relied on both unidimensional and multidimensional divisions [62,63,64,65]. In this study, the perceived value was defined as consumers’ measures of the benefits and costs of shopping through an FFEP.

Consumers develop their preference for a brand based on different sorts of information, leading to a further enhancement of the brand’s perceived value [66]. Consequently, as consumers acquire more knowledge about a brand, they generally treat it as a better value provider [67]. When consumers are not acquainted with a particular product, they may opt for a popular brand instead of another, due to the psychological benefits they may obtain from its popularity [68]. Therefore, when purchasing fresh food, consumers may opt for a well-known FFEP to experience the psychological benefits associated with these platforms. Strong brand images usually generate high levels of perceived value [69,70], especially since successful brand images create value for customers [71]. Similarly, research on internet marketing suggests that a reliable and satisfactory brand enhances the perceived value of products [72]. The following hypotheses are deduced from the above analysis:

**Hypothesis** **2a** **(H2a).** *Brand awareness of the FFEP positively affects consumer perceived value.*

**Hypothesis** **2b** **(H2b).** *The brand image of the FFEP positively affects consumer perceived value.*

#### 2.1.3. Brand Knowledge and Brand Trust

Trust in a product is formed over time based on a series of interactions and observations [73,74], which are influenced by consumers’ direct and indirect association with a brand [75]. As such, brand trust is typically studied from the subjective psychological perspective of consumers. In early studies, brand trust was defined as the consumers’ subjective emotions toward a company brand upon interaction with it [76]. Subsequent research provided new definitions with differing emphases. For instance, scholars are interested in the impact of perceived risk and define the concept as consumers’ confidence in the reliability of a brand and their behavioral intentions even in the presence of risks [77]. Other researchers, based on consumer preferences, have defined the concept as consumers’ psychological expectations regarding the reliability, consistency, and other attributes of all products of a brand [78]. This kind of trust comes from consumers’ acquisition of brand product knowledge and their existing emotional connection with the brand [79,80]. One of the ultimate objectives of corporate marketing is to establish a solid connection between brands and consumers, with consumer trust being its base [81]. Thus, in the context of relationship marketing, trust has not only been conceptualized as a crucial factor for marketing success [82], but it has also emerged as a popular topic in online marketing [83,84].

Trust is a mental process [85,86,87], and brand trust is gradually established over time as consumers accumulate experience and knowledge about a brand. Therefore, brand knowledge is the fundamental element for forming brand trust [88]. Without further comparison information, brands can influence consumers’ online choices [89]. High brand awareness results in a positive brand image, which, in turn, leads to better brand trust [90]. Consumers might trust a platform due to its brand awareness when purchasing fresh food online. Additionally, research demonstrates that if consumers believe that a product has a good brand image, they tend to consider it a quality product [91]. Therefore, an FFEP with an excellent image is better positioned to enhance consumers’ trust. The following hypotheses are deduced from the above analysis:

**Hypothesis** **3a** **(H3a).** *Brand awareness of the FFEP positively affects consumer brand trust.*

**Hypothesis** **3b** **(H3b).** *The brand image of the FFEP positively affects consumer brand trust.*

#### 2.1.4. Perceived Value and Purchase Intention

Customer perceived value serves as a bridge between the consumer and their consumption behavior, connecting their psychological activities with the consumer behavior process [23], and is a crucial criterion for evaluating their choices [92]. Numerous studies have found that perceived consumer value has a significant impact on consumer behavior regarding both offline purchases and online shopping. More specifically, higher perceived value leads to stronger purchase intention [93,94,95,96]. Although this advantageous relationship has been well studied, the hypothesis is included in this paper for the sake of completeness for this study and consistency of subsequent studies. The following hypothesis is deduced from the above analysis:

**Hypothesis** **4** **(H4).** *The consumer-perceived value of the FFEP positively influences the purchase intention of fresh food.*

#### 2.1.5. Brand Trust and Purchase Intention

Trust is a significant predictor of consumer purchase behavior [97]. If consumers are uncertain about or attentive to a brand’s motivation and the brand shows an unclear tendency, their purchase intention decreases [98]. Brand trust can also positively impact purchase intentions and behavior by reducing the psychological effect of uncertainty risk and incentivizing consumers to make a purchase [99]. The same applies to online purchases [100,101]. Consumers tend to select brands that offer a sense of security during online transactions, and the ability of a brand to provide this sense of security is a crucial factor in establishing brand trust [102]. Thus, consumers’ trust in an FFEP can boost their intention to buy fresh food. The following hypothesis is deduced from the above analysis:

**Hypothesis** **5** **(H5).** *Consumer brand trust in the FFEP positively affects the purchase intention of fresh food.*

#### 2.1.6. Perceived Value and Brand Trust

A brand needs to reflect trust, and consumers usually appreciate good brands because they represent high-quality products and enhance consumer confidence and security [27]. Previous research has demonstrated that meeting consumers’ expectations for product value can increase their trust in a brand [103,104]. High levels of perceived value can also increase consumers’ confidence in a product after the purchase [105]. When consumers believe they have received excellent product or service value, their satisfaction and trust in the brand may increase [106]. Therefore, the value consumers receive from the platform can enhance their trust in the brand. The following hypothesis is deduced from the above analysis:

**Hypothesis** **6** **(H6).** *The consumer-perceived value of the FFEP positively affects consumer brand trust.*

#### 2.1.7. Serial Mediation Effect of Brand Trust and Perceived Value

This study considers perceived value and brand trust to have a serial mediation impact between brand knowledge and consumer purchase intention, according to the hypotheses outlined above. The behavior of consumers purchasing fresh food online is influenced by brand awareness and brand image, which can lower their knowledge of unknown risks and encourage them to subjectively discern and judge the product’s value (based on the derivation of H2a and H2b) [68,70]. This value image can increase consumers’ trust in the platform (based on the derivation of H6) and further trigger their purchase intention (based on the derivation of H5) [100,101,106]. The integration of the above hypotheses suggests that brand awareness and image can shape consumers’ perception of value [107], gradually strengthening their trust in the FFEP and ultimately positively impacting their intention to purchase fresh food [103,108,109,110]. The following hypotheses are deduced from the above analysis:

**Hypothesis** **7a** **(H7a).** *Perceived value plays a mediating effect between brand awareness and consumer purchase intention.*

**Hypothesis** **7b** **(H7b).** *Perceived value plays a mediating effect between brand image and consumer purchase intention.*

**Hypothesis** **7c** **(H7c).** *Brand trust plays a mediating effect between brand awareness and consumer purchase intention.*

**Hypothesis** **7d** **(H7d).** *Brand trust plays a mediating effect between brand image and consumer purchase intention.*

**Hypothesis** **7e** **(H7e).** *Perceived value and brand trust play a serial mediating effect between brand awareness and consumer purchase intention.*

**Hypothesis** **7f** **(H7f).** *Perceived value and brand trust play a serial mediating effect between brand image and consumer purchase intention.*

### 2.2. Research Model

**Figure 1:**
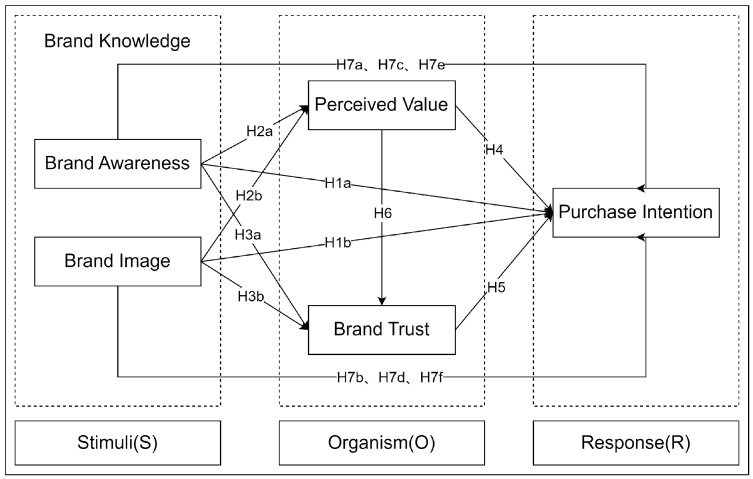
Model of the effect of FFTP brand knowledge on fresh food purchase intention.

The SOR theory, widely applied in online purchase research [111], holds great significance in establishing the model framework of this study and understanding the relationships between variables. According to this theory, “S” denotes external stimuli, “O” represents the internal state influenced by stimuli, and “R” signifies behaviors influenced by the stimuli [112]. Drawing from insights from previous studies [113,114,115], this research considers brand knowledge (brand awareness, brand image) as the stimulus “S”, perceived value and brand trust as the organism “O”, and purchase intention as the response “R”. Based on this analysis and assumption, the research model for this study was derived, and is illustrated in Figure 1.

## 3. Methodology

### 3.1. Data Collection

The questionnaire items were designed based on previous questionnaires that have been effectively used in relevant studies, which also have been further adapted for this study. Before the formal survey, a pilot survey of 30 consumers was conducted to optimize and modify the questionnaire’s language for improved readability. From February 2023 to March 2023, an Internet-based questionnaire was used for data collection, and all respondents were recruited from wjx.cn, a professional survey website. wjx.cn serves as the most extensive questionnaire survey website in China. It enables both wjx.cn users and non-users to effortlessly complete the questionnaire on mobile terminals without the involvement of any restrictions. The target area of the questionnaire was mainland China, and the survey was primarily promoted through various platforms, including WeChat chat, WeChat friends circle, QQ, and QQ space. To ensure data accuracy, only consumers with prior experience in purchasing fresh food from platforms were surveyed and a duplicate question was added about the number of times per month using the FFEP. Inconsistent questionnaires were later removed during the data cleaning to ensure questionnaire quality. All the participants who completed the questionnaire received 3 RMB as a token of appreciation. The 558 questionnaires collected were screened, and 83 unreliable questionnaires were removed, leaving 475 questionnaires for data analysis. From a data perspective, the sample size of 475 met the requirement of at least 10 data points corresponding to each measurement item (20 measurement items) [116].

Furthermore, it is worth mentioning that this study employed a self-selected (volunteer) sampling method. Self-selected (volunteer) sampling possesses the following characteristics when compared to other sampling methods: first of all, convenience. Additionally, participants volunteered to take part, which significantly eased the process of questionnaire collection and facilitated the implementation of the study. Secondly, it ensured high participation rates. As participants voluntarily opted to partake in the study, they likely exhibited greater interest and motivation leading to enhanced participation. Thirdly, the approach fostered authenticity and spontaneity in the results. By employing self-selected sampling, the study’s outcomes became more reliable as participants’ involvement was voluntary rather than coerced. This aspect enabled the reflection of genuine attitudes, behaviors, and opinions.

### 3.2. Measures

The questionnaire consisted of 20 scale questions and 6 questions on respondent characteristics, as shown in Table 1 and Appendix A. The scale questions were measured using a 7-point Likert scale ranging from 1 (strongly disagree) to 7 (strongly agree).

Bollen and Lennox emphasized the importance of considering the directionality between observed variables and latent variables in designing the scale to enhance the structural validity of the measurement [117]. Observations of the variables in this study revealed the following characteristics: All the observed variables corresponding to the same latent variable reflected common themes. Each observed variable was determined by the latent variable to which it belongs, and the observed variables of the same latent variable exhibited high correlations. Based on studies by Diamantopoulos et al., Edwards et al., and MacKenzie et al. [118,119,120], the variables in this study are reflective models. Confirmatory Tetrad Analysis (CTA-PLS) was utilized to determine the directionality between the observed and latent variables because the latent variables in this study consisted of four observed variables [121]. The criterion validation depends on whether the tetrads values are significantly different from zero [122]. In the validation results, the differences between the observed variables and the latent variables are found to be statistically insignificant, with the presence of zeros falling within the lower-adjusted confidence interval (CI) and the upper-adjusted CI (see Appendix B). Therefore, the observed variables exhibit high and exchangeable correlation, reconfirming that the variables in this study are reflective models [123].

**Table 1:** Measurement.

Constructs	Items	Sources
Brand Awareness (BA)	I can easily distinguish this brand from competing brands.	Dabbous et al. Tong et al. [124,125]
	The brand identity of this platform comes to mind quickly.	
	My friends are also familiar with this e-commerce brand.	
	I am more familiar with this platform compared to others.	
Brand Image (BI)	I believe this platform holds a prominent position in its industry.	Belén del Río et al. [126]
	I believe the products on this platform are of reliable quality.	
	I believe this platform has an excellent reputation.	
	I believe that using this platform is a status symbol.	
Perceived Value (PV)	I think the quality of the products/services on this platform is very good.	Liu et al. [64]
	I think the platform provides an enjoyable shopping experience.	
	I find this platform offers excellent value for money.	
	I think my shopping on this platform is well regarded by others.	
Brand Trust (BT)	I think I can trust this platform.	Munuera-Aleman et al. [127]
	I think the platform meets my expectations.	
	I feel confident and assured about my purchases on this platform.	
	I believe any problems I encounter with this platform will be resolved satisfactorily.	
Purchase Intention (PI)	I would first consider buying fresh food from this platform.	Peng et al. [23]
	I am eager to purchase fresh food from this platform.	
	I think the fresh food available on this platform is worth buying.	
	I am happy to recommend my friends to buy fresh food from this platform.	

## 4. Results

This study used SPSS 26.0 (IBM, Armonk, NY, USA) software for respondent characterization of the study sample, and SmartPLS4 (SmartPLS GmbH, Oststeinbek, Germany) software for data analysis and hypothesis validation. SmartPLS considers the feasibility of all paths in the model, which can make it easier for researchers to obtain results [128]. The data were analyzed in two steps using the SmartPLS algorithm [122]. Firstly, the PLS-SEM algorithm was utilized to evaluate the measurement model. Secondly, the structural model was evaluated using the bootstrapping and PLS-prediction algorithms [129].

### 4.1. Demographic Profile

The results of the descriptive statistical analysis of the sample using SPSS 26.0 software are presented in Table 2. The data showed a higher percentage of female respondents (61.7%) compared to male respondents (38.3%). This finding aligns with the view that women are the main decision-makers in food purchases [130]. In terms of age characteristics, respondents aged 21–30 and 31–40 accounted for 47.8% and 36.2% of the overall sample, which relates to the higher spending power of this age group and largely corresponds to the age distribution of the online shopping population in China [131]. Concerning education level, most consumers in the sample have obtained a Bachelor’s degree (58.7%). With regard to the frequency of using FFEP monthly, most consumers used them less than three times (45.9%) or between 3 and 6 times (38.1%). These findings indicate that the sample for this questionnaire survey is representative of Chinese online shoppers and has wide coverage.

### 4.2. Reliability and Validity Analysis

This study assessed the reliability and validity of the measurement model using Quality Criteria in the PLS-SEM algorithm. The results presented in Table 3 indicate that both Cronbach’s Alpha coefficient (0.833–0.868) and CR values (0.889–0.910) exceeded the recommended threshold of 0.7 [132,133]. Additionally, the external factor loadings (0.796–0.885) and Average Variance Extracted values (AVE, 0.666–0.716) were higher than the standard values of 0.6 and 0.5, respectively [132,134]. The discriminant validity analysis demonstrated that the square root of the AVE for each variable is greater than the absolute value of the correlation coefficient between those variables and other variables, as shown in Table 4. Furthermore, all Heterotrait–Monotrait Ratio (HTMT) values exceeded the accepted limit of 0.9 [135], as shown in Table 5. As a result, this study’s measurement model exhibited high reliability, convergent validity, and discriminant validity.

### 4.3. Collinearity Diagnostics

Prior to the path analysis, the presence of multicollinearity problems was checked by the VIF value of the inner model matrix, as shown in Table 6. The data show that the VIF values (1.081–1.958) are all below the recommended threshold of 5 [134]. Therefore, the co-collinearity problem does not lead to substantial errors in determining the structural model’s path coefficients, and further research can be conducted.

### 4.4. Hypothesis Validation

**Figure 2:**
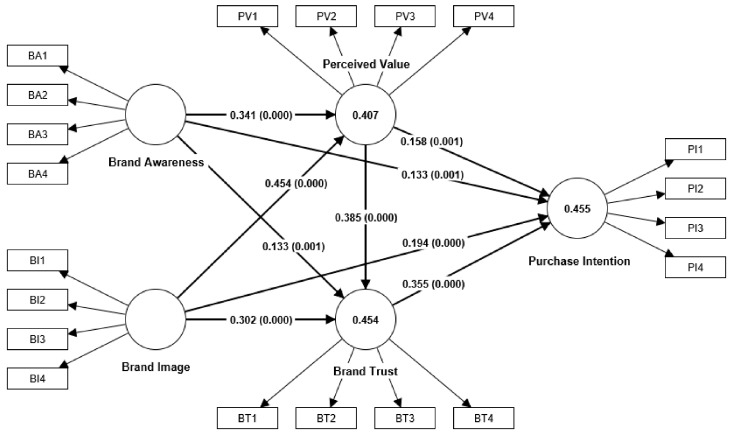
Analytical result of the model.

The structural model was tested using the bootstrapping method, as shown in Table 7 and Figure 2. According to previous studies, the path passed the significance test when *t* > 1.96 and *p* < 0.05 [136]. The results of path analysis show that BA (β = 0.133, *t* = 3.382, *p* = 0.001) and BI (β = 0.194, *t* = 4.624, *p* = 0.000) have a significant positive impact on PI. Hypotheses H1a and H1b are supported. BA (β = 0.341, *t* = 9.481, *p* = 0.000) and BI (β = 0.454, *t* = 12.825, *p* = 0.000) have a significant positive impact on PV. Hypotheses H2a and H2b are supported. BA (β = 0.133, *t* = 3.442, *p* = 0.001) and BI (β = 0.302, *t* = 7.076, *p* = 0.000) have a significantly positive impact on BT. Hypotheses H3a and H3b are supported. PV (β = 0.158, *t* = 3.441, *p* = 0.001) has a significant positive impact on PI. Hypothesis H4 is supported. BT (β = 0.355, *t* = 7.626, *p* = 0.000) has a significant positive impact on PI. Hypothesis H5 is supported. PV (β = 0.385, *t* = 8.861, *p* = 0.000) has a significant positive effect on BT. Hypothesis H6 is supported. In the hypothesis where testing mediation, this study followed Preacher et al.’s suggestion and tested its mediating effect by implementing the bootstrapping method. The results found a significant mediating effect of PV between BA and PI (β = 0.054, *t* = 3.204, *p* = 0.001) and between BI and PI (β = 0.072, *t* = 3.291, *p* = 0.001). Hypotheses H7a and H7b are supported. BT has a significant mediating effect between BA and PI (β = 0.047, *t* = 3.250, *p* = 0.001) and between BI and PI (β = 0.107, *t* = 4.983, *p* = 0.000). Hypotheses H7c and H7d are supported. PV and BT have a significant serial mediation effect between BA and PI (β = 0.047, *t* = 4.661, *p* = 0.000) and between BI and PI (β = 0.062, *t* = 5.313, *p* = 0.000). Hypotheses H7e and H7f are supported. 

### 4.5. Explanatory Power and Predictive Power of the Model

The R^2^ and Q^2^ values of the model were examined by the PLS-SEM algorithm and the PLS-Predict algorithm, which were used to assess the model’s explanatory power and predictive ability. As shown in Table 8, the R^2^ values of all variables were higher than the recommended threshold of 0.25 [137], and the Q^2^ values were higher than the recommended threshold of 0 [138]. Therefore, the model in this study has strong explanatory and predictive power [139].

## 5. Discussion

While brand knowledge is known to be an essential factor affecting consumer behavior, its role in FFEP and purchase intentions remain unclear. Therefore, the present study explores how brand knowledge impacts purchase intentions in FFEP. Additionally, the study proposes to use perceived value and brand trust as mediating variables in the model, considering their influence on purchase intentions. Moreover, this study verified the joint effect of these two variables on customers’ online purchases of fresh food.

Firstly, brand awareness and brand image significantly positively affected the online purchase intention of fresh food. This result is similar to the outcomes of previous studies based on online shopping [40,44]. Differently, brand awareness in this study focuses on the FFEP rather than the product itself. Similarly, the focus of this study is on the direct role of brand image rather than its mitigating effect on online shopping risk, which expands the scope of brand image research. Furthermore, the findings from certain previous studies are in line with the results of this paper [140,141], suggesting that a stronger accumulation of consumers’ memories and associations with the FFEP brand positively influences their inclination to make a purchase. The data also reveal that consumers deem brand image more important, making it more influential in their purchase intentions. This demonstrates the importance of brand image when evaluating purchase intentions. It also implies that consumers value the reputation, product quality, and status symbols of the platform more than the popularity of the FFEP. Therefore, the halo effect from the brand image can better boost consumers’ purchase intentions.

Secondly, brand awareness and brand image significantly positively impact perceived value. This is contrary to the conclusions of previous studies but follows the conclusions made by Dodds et al. [70,142]. However, there are two differences between Dodds et al.’s conclusions and this study. Firstly, this study focuses on online shopping behavior, not on offline shopping. Secondly, this study uses awareness and image as the entry point for analyzing brands rather than from a name perspective. Among various online studies, the study of Abu ELSamen is consistent with the results of this study, which suggests that the more consumers are acquainted with FFEP and come across it more often, the more they value FFEP [107]. Moreover, when comparing the path coefficients of the antecedents of perceived value, brand image was the most powerful predictor of perceived value. This shows that when FFEPs have a brand image they recognize, they are more likely to generate perceived value.

Thirdly, brand awareness and brand image significantly positively affect brand trust. This result is consistent with prior research on a screen golf game system conducted in Korea [90]. This not only indicates that the research findings can be applied to FFEP but also suggests that consumers’ understanding of FFEP can reduce their concerns about the associated risks and enhance their level of brand trust in FFEP. Furthermore, research suggests that brand image has a bigger influence on brand trust generation than brand awareness. This result may be because brand image reflects authoritative attributes such as reputation, product quality, and status. The authority bias effect holds that the greater the authority of a person, the more quickly their words or actions are believed to be correct by others.

Additionally, brand trust and perceived value are recognized as critical elements of the FFEP influencing consumer purchase intentions. The first hypothesis was also supported by prior research on e-commerce purchase intentions [23], which suggests that the higher the value consumers perceive from the FFEP brand, the more likely they are to have purchase intentions. However, when it comes to the relationship between trust and purchase intention, previous studies have presented contrasting results to the findings of this paper [143]. Previous studies have suggested that experienced online consumers are less concerned about security, leading to trust having an insignificant effect on purchase intention. However, many of the fresh food e-commerce platforms are relatively new and have been launched recently. Moreover, the characteristics of fresh food, such as perishability, may trigger a higher level of safety concern among consumers, thus influencing their trust in these platforms. Among the factors influencing purchase intention, brand trust has the greatest impact. This highlights the importance of brand trust’s presence when consumers make use of an FFEP. This finding aligns with Simmel’s theory of trust, which argues that society begins with interaction and that exchange is the main form of interaction, especially monetary-mediated exchange, which cannot occur without trust [144].

Furthermore, perceived value has a significant positive impact on brand trust. This indication is similar to the results of a study on hotels [106], according to which the higher the value consumers perceive in the FFEP brand, the higher their confidence and validation of the FFEP brand. In addition, according to the data gathered from this study, the most powerful predictor of all antecedents of brand trust is perceived value. This suggests that the perception of FFEP value is more likely to drive a psychological response of trust in consumers than brand awareness and brand image.

Furthermore, there are significant mediating effects of perceived value and brand trust between the components of brand knowledge and consumers’ purchase intention. In previous studies, no identical hypotheses were available for reference, but this extends the previous research on the mediating role of perceived value and brand trust [145,146], further validating the significant role of perceived value and brand trust in enhancing consumers’ purchase intention. More specifically, brand awareness and image determine the degree to which consumers remember and recognize FFEP, and the more vivid these memories and recognition are, the stronger the perceived level of value and trust will be. This heightened perception of value and trust, in turn, reinforces consumers’ purchase intentions for fresh food. This finding further enriches our understanding of the critical role of these two variables in shaping buying behavior in the e-commerce context.

Finally, the analysis reveals a significant serial mediation effect of perceived value and brand trust between brand knowledge dimensions and purchase intention. Although identical hypotheses were not found in previous studies for reference, this study extends the theoretical framework concerning perceived value and brand trust [147], contributing to the validation of the inherent interplay between perceived value, brand trust, and consumer purchase intention. More specifically, when consumers have a strong recall and recognition of an FFEP, it tends to increase their perceived value. This perception of value can raise their expectations and confidence in the FFEP, ultimately enhancing their purchase intention. Therefore, the research framework proposed in this study, which includes brand knowledge, perceived value, brand trust, and purchase intention is valid and supported by the findings.

## 6. Conclusions

This study has several critical theoretical implications for the current research. Firstly, past research on FFEPs and consumer behavior has focused on developing products and platforms. Yet, this study contributes to the recent research on consumer behavior in the context of FFEPs by highlighting the connections between brand knowledge, perceived value, brand trust, and purchase intention in the FFEP domain using a model framework. Furthermore, past research on the relationship between brand knowledge and consumer behavior ignored the influence of perceived value and brand trust. By verifying the mediating effects of these two variables, this study enhances and broadens the understanding of the relationship between brand knowledge and consumer behavior in FFEP. Based on these findings, we propose the following recommendations.

Firstly, it is recommended to enhance the advertising of FFEPs. Advertising serves as the most direct and efficient way for consumers to engage with a brand. Therefore, advertising can constantly strengthen consumers’ recall of the brand, thus it improves brand awareness.

Secondly, it is recommended to enhance the visual image design of the FFEP. This can be achieved by optimizing the design of the platform logo, layout, brand colors, and mascot using visual design. Highlight the brand personality to enhance consumer recognition of the platform and thus enhance brand awareness.

Moreover, prioritizing product quality and reputation plays a vital role in bolstering the platform’s position within the industry. By rigorously controlling quality and maintaining a positive reputation, consumers perceive the platform as reliable and a symbol of status, consequently elevating the brand image.

Additionally, enhancing the service quality of the platform is essential. Establishing a comprehensive mechanism to address sales issues instils confidence in consumers that the platform can effectively handle any potential problems during the purchasing process. This fosters trust and high expectations toward the platform, thereby enhancing consumer-perceived value and further elevating brand trust.

## 7. Limitations and Future Research

Like any other type of research, this study inevitably has some limitations. First of all, this study gathered 475 valid questionnaires from China using a self-selected (volunteer) sampling method, which may have introduced sampling bias. It is possible that individuals who volunteered to participate in the survey possess distinct characteristics from non-volunteer participants, thereby resulting in a somewhat biased sample. Additionally, the sample size remained limited, and while efforts were made to include various fresh food e-commerce platforms, it may not encompass all platforms available. Consequently, the findings could be subject to limitations concerning the representativeness of the sample. To enhance the generalizability of the results, future studies will employ random sampling and distribute questionnaires to consumers from diverse countries.

Furthermore, this study did not consider the influence of certain other factors (such as familiarity with online shopping and mobile features) on purchase intention; for example, as mentioned earlier, consumers’ familiarity with online shopping behavior affects their need for security, which reduces the influence of trust on purchase intention. Therefore, future research should include relevant variables such as familiarity with online shopping and mobile features to gain a more comprehensive understanding. Finally, although this study addressed FFEPs’ brand knowledge, it solely focused on the FFEPs’ own brand knowledge while disregarding the potential brand knowledge associated with the parent company of the FFEP. As a result, in future research, FFEPs should be categorized to compare the differences and impact of both the platform’s brand knowledge and the brand knowledge attached to the present company.

## Figures and Tables

**Table 2:** Descriptive statistics (N = 475).

	Items	Frequency	Proportion
Gender	Male	182	38.3%
	Female	293	61.7%
Age (in years)	<20	12	2.5%
	21–30	227	47.8%
	31–40	172	36.2%
	>41	64	13.5%
Education	High school and below	38	8.0%
	College	85	17.9%
	Undergraduate	279	58.7%
	Graduate student	73	15.4%
Job	Career unit	41	8.6%
	State-owned enterprises	125	26.3%
	Private enterprises	187	39.4%
	Student	86	18.1%
	Others	36	7.6%
Monthly income (RMB/Yuan)	<3000	45	9.5%
	3000–6000	158	33.3%
	6000–9000	120	25.3%
	9000–12,000	97	20.4%
	>12,000	55	11.6%
Number of monthly uses	<3	218	45.9%
	3–6	181	38.1%
	7–10	65	13.7%
	>10	11	2.3%

**Table 3:** Reliability and validity analysis.

Constructs	Item	Factor Loadings	Cronbach’s Alpha	CR	AVE
Brand Awareness (BA)	BA1	0.830	0.859	0.904	0.703
	BA2	0.885			
	BA3	0.824			
	BA4	0.812			
Brand Image (BI)	BI1	0.796	0.846	0.896	0.684
	BI2	0.807			
	BI3	0.863			
	BI4	0.841			
Perceived Value (PV)	PV1	0.853	0.862	0.906	0.707
	PV2	0.837			
	PV3	0.831			
	PV4	0.842			
Brand Trust (BT)	BT1	0.856	0.868	0.910	0.716
	BT2	0.859			
	BT3	0.835			
	BT4	0.835			
Purchase Intention (PI)	PI1	0.797	0.833	0.889	0.666
	PI2	0.859			
	PI3	0.805			
	PI4	0.802			

**Table 4:** Discriminant validity (FORNELL).

	BA	BI	PV	BT	PI
Brand Awareness (BA)	0.838				
Brand Image (BI)	0.274	0.827			
Perceived Value (PV)	0.465	0.547	0.841		
Brand Trust (BT)	0.395	0.549	0.612	0.846	
Purchase Intention (PI)	0.400	0.512	0.543	0.611	0.816

**Table 5:** Discriminant validity (HTMT).

	BA	BI	PV	BT	PI
Brand Awareness (BA)					
Brand Image (BI)	0.320				
Perceived Value (PV)	0.540	0.638			
Brand Trust (BT)	0.454	0.638	0.707		
Purchase Intention (PI)	0.471	0.606	0.636	0.715	

**Table 6:** VIF value of the inner model matrix.

	BA	BI	PV	BT	PI
Brand Awareness (BA)			1.081	1.277	1.309
Brand Image (BI)			1.081	1.428	1.596
Perceived Value (PV)				1.686	1.958
Brand Trust (BT)					1.833
Purchase Intention (PI)					

**Table 7:** Hypothesis testing.

Hypotheses	β	SD	T	*p*	LLCI	ULCI	Decision
BA -> PI	0.133	0.039	3.382	0.001	0.055	0.208	Supported
BI -> PI	0.194	0.042	4.624	0.000	0.109	0.276	Supported
BA -> PV	0.341	0.036	9.481	0.000	0.271	0.413	Supported
BI -> PV	0.454	0.035	12.825	0.000	0.384	0.523	Supported
BA -> BT	0.133	0.039	3.442	0.001	0.056	0.209	Supported
BI -> BT	0.302	0.043	7.076	0.000	0.219	0.386	Supported
PV -> PI	0.158	0.046	3.441	0.001	0.068	0.248	Supported
BT -> PI	0.355	0.047	7.626	0.000	0.263	0.446	Supported
PV -> BT	0.385	0.043	8.861	0.000	0.300	0.469	Supported
BA -> PV -> PI	0.054	0.017	3.204	0.001	0.023	0.089	Supported
BI -> PV -> PI	0.072	0.022	3.291	0.001	0.031	0.116	Supported
BA -> BT -> PI	0.047	0.015	3.250	0.001	0.020	0.076	Supported
BI -> BT -> PI	0.107	0.022	4.983	0.000	0.069	0.152	Supported
BA -> PV -> BT -> PI	0.047	0.010	4.661	0.000	0.029	0.069	Supported
BI -> PV -> BT -> PI	0.062	0.012	5.313	0.000	0.041	0.087	Supported

**Table 8:** R^2^ value and Q^2^ value.

	R^2^	Q^2^ Predict
Perceived Value (PV)	0.407	0.401
Brand Trust (BT)	0.454	0.360
Purchase Intention (PI)	0.455	0.327

## Data Availability

The data supporting the findings of the current study are available from the corresponding author.

## Appendix A. Formal Questionnaire

Part I Demographic measures

1. Your gender: a. Male b. Female
2. Your age: a. Less than 20 years old b. 21–30 years old c. 31–40 years old d. Greater than 41 years old
3. Education level: a. High school and below b. College c. Undergraduate d. Graduate student
4. Your Occupation: a. Career unit b. State-owned enterprises c. Private enterprises d. Student e. Others
5. Your average monthly income (RMB/Yuan): a. Less than 3000, b. 3000–6000, c. 6000–9000, d. 9000–12,000, e. More than 12,000
6. Number of uses per month: a. Less than 3, b. 3–6, c. 7–10, d. More than 10

Part II Measurement scales

- Brand Awareness
  1. I can easily distinguish this brand from competing brands.
  2. The brand identity of this platform comes to mind quickly.
  3. My friends are also familiar with this e-commerce brand.
  4. I am more familiar with this platform compared to others.
- Brand Image
  1. I believe this platform holds a prominent position in its industry.
  2. I believe the products on this platform are of reliable quality.
  3. I believe this platform has an excellent reputation.
  4. I believe that using this platform is a status symbol.
- Perceived Value
  1. I think the quality of the products/services on this platform is very good.
  2. I think the platform provides an enjoyable shopping experience.
  3. I find this platform offers excellent value for money.
  4. I think my shopping on this platform is well-regarded by others.
- Brand Trust
  1. I think I can trust this platform.
  2. I think the platform meets my expectations.
  3. I feel confident and assured about my purchases on this platform.
  4. I believe any problems I encounter with this platform will be resolved satisfactorily.
- Purchase Intention
  1. I would first consider buying fresh food from this platform.
  2. I am eager to purchase fresh food from this platform.
  3. I think the fresh food available on this platform is worth buying.
  4. I am happy to recommend my friends to buy fresh food from this platform.

Note: The scale questions were measured using a 7-point Likert scale ranging from 1 (strongly disagree) to 7 (strongly agree).

## Appendix B

**Table A1:** Confirmatory Tetrad Analysis (CTA-PLS).

Matrix	T Statistics	*p* Values	CI Low Adj.	CI Up Adj.
1: BA1,BA2,BA3,BA4	0.772	0.440	−0.327	0.143
2: BA1,BA2,BA4,BA3	0.513	0.608	−0.273	0.158
1: BI1,BI2,BI3,BI4	1.233	0.218	−0.097	0.437
2: BI1,BI2,BI4,BI3	1.089	0.276	−0.117	0.413
1: PV1,PV2,PV3,PV4	1.322	0.186	−0.420	0.081
2: PV1,PV2,PV4,PV3	1.657	0.098	−0.446	0.035
1: BT1,BT2,BT3,BT4	0.142	0.887	−0.229	0.267
2: BT1,BT2,BT4,BT3	0.433	0.665	−0.193	0.306
1: PI1,PI2,PI3,PI4	0.215	0.830	−0.208	0.262
2: PI1,PI2,PI4,PI3	0.228	0.819	−0.187	0.236
